# Modulating the Activity of the Human Organic Cation Transporter 2 Emerges as a Potential Strategy to Mitigate Unwanted Toxicities Associated with Cisplatin Chemotherapy

**DOI:** 10.3390/ijms25052922

**Published:** 2024-03-02

**Authors:** Anna Hucke, Marta Kantauskaite, Tim N. Köpp, Christoph A. Wehe, Uwe Karst, Pavel I. Nedvetsky, Giuliano Ciarimboli

**Affiliations:** 1 Experimental Nephrology, Department of Internal Medicine D, University Hospital Münster, 48149 Münster, Germany; a_huck01@uni-muenster.de (A.H.); marta.kantauskaite@med.uni-duesseldorf.de (M.K.); tim.koepp123@gmail.com (T.N.K.); nedvetsky@uni-muenster.de (P.I.N.); 2 Institute of Physiology I, University of Münster, 48149 Münster, Germany; 3 Klinik für Nephrologie, Universitätsklinikum Düsseldorf, 40225 Düsseldorf, Germany; 4 Institute of Inorganic and Analytical Chemistry, University of Münster, 48149 Münster, Germany; christoph.wehe@thermofisher.com (C.A.W.); uk@uni-muenster.de (U.K.)

**Keywords:** cisplatin, toxicity, transport, organic cation transporter, regulation, competition, protection

## Abstract

Cisplatin (CDDP) stands out as an effective chemotherapeutic agent; however, its application is linked to the development of significant adverse effects, notably nephro- and ototoxicity. The human organic cation transporter 2 (hOCT2), found in abundance in the basolateral membrane domain of renal proximal tubules and the Corti organ, plays a crucial role in the initiation of nephro- and ototoxicity associated with CDDP by facilitating its uptake in kidney and ear cells. Given its limited presence in cancer cells, hOCT2 emerges as a potential druggable target for mitigating unwanted toxicities associated with CDDP. Potential strategies for mitigating CDDP toxicities include competing with the uptake of CDDP by hOCT2 or inhibiting hOCT2 activity through rapid regulation mediated by specific signaling pathways. This study investigated the interaction between the already approved cationic drugs disopyramide, imipramine, and orphenadrine with hOCT2 that is stably expressed in human embryonic kidney cells. Regarding disopyramide, its influence on CDDP cellular transport by hOCT2 was further characterized through inductively coupled plasma isotope dilution mass spectrometry. Additionally, its potential protective effects against cellular toxicity induced by CDDP were assessed using a cytotoxicity test. Given that hOCT2 is typically expressed in the basolateral membrane of polarized cells, with specific regulatory mechanisms, this work studied the regulation of hOCT2 that is stably expressed in Madin–Darby Canine Kidney (MDCK) cells. These cells were cultured in a matrix to induce the formation of cysts, exposing hOCT2 in the basolateral plasma membrane domain, which was freely accessible to experimental solutions. The study specifically tested the regulation of ASP^+^ uptake by hOCT2 in MDCK cysts through the inhibition of casein kinase II (CKII), calmodulin, or p56*^lck^* tyrosine kinase. Furthermore, the impact of this manipulation on the cellular toxicity induced by CDDP was examined using a cytotoxicity test. All three drugs—disopyramide, imipramine, and orphenadrine—demonstrated inhibition of ASP^+^ uptake, with IC_50_ values in the micromolar (µM) range. Notably, disopyramide produced a significant reduction in the CDDP cellular toxicity and platinum cellular accumulation when co-incubated with CDDP. The activity of hOCT2 in MDCK cysts experienced a significant down-regulation under inhibition of CKII, calmodulin, or p56*^lck^* tyrosine kinase. Interestingly, only the inhibition of p56*^lck^* tyrosine kinase demonstrated the capability to protect the cells against CDDP toxicity. In conclusion, certain interventions targeting hOCT2 have demonstrated the ability to reduce CDDP cytotoxicity, at least in vitro. Further investigations in in vivo systems are warranted to ascertain their potential applicability as co-treatments for mitigating undesired toxicities associated with CDDP in patients.

## 1. Introduction

Cisplatin (cis-dichlorodiammine platinum (II), CDDP) is an important component of cancer chemotherapy, which is used as a single agent or in combination with other anti-neoplastic drugs, radiotherapy, and immunotherapy for the treatment of various types of cancer [1,2]. A significant challenge associated with CDDP-based chemotherapy is the occurrence of significant undesired toxic effects, notably nephro- and ototoxicity. These side effects not only hinder the effectiveness of cancer treatment but also adversely impact the quality of life of cancer survivors. Therefore, it is imperative to understand the molecular mechanisms underlying CDDP toxicity. This understanding is crucial for the development of effective therapeutic strategies aimed at mitigating these undesired toxicities, all while preserving the anticancer efficacy of the treatment. As described in the following, CDDP exerts its toxic effects intracellularly. Upon entering the cell, CDDP undergoes aquation, leading to the formation of aquations [3]. These aquations subsequently interact with the adenine or guanine bases of DNA molecules, creating intra- and interstrand adducts [4]. This molecular interaction disrupts normal DNA replication and transcription processes, ultimately triggering cellular apoptosis [5]. Hence, the rate of CDDP cellular entry plays a pivotal role in determining the extent of CDDP-induced cellular toxicity. CDDP is a hydrophilic drug, whose cellular uptake has been elucidated to occur through passive diffusion processes [6] or be facilitated by specific membrane transporters, notably the copper transporter 1 (Ctr1) [7] and organic cation transporters (OCTs), mainly the OCT2 [8]. The expression of Ctr1 in cancer cells appears to be a critical factor in facilitating CDDP uptake and enhancing the anti-tumor efficacy of the drug [9]. Conversely, the distinct organ distribution of OCT2 emerges as a decisive factor in the manifestation of specific adverse effects associated with CDDP treatment, including nephrotoxicity [10,11,12] and ototoxicity [10]. Hence, targeting OCT2 could serve as a viable strategy for developing protective interventions aimed at mitigating undesired CDDP toxicity in the kidneys and the auditory cells. This approach aims to preserve the antineoplastic efficacy of CDDP while minimizing adverse effects in these specific organs [13]. Strategies for targeting OCT2 to alleviate undesired CDDP toxicity may include employing specific OCT2 inhibitors to compete for the transporter or implementing rapid regulation of OCT2 activity by cellular signaling pathways. These interventions could be administered simultaneously with CDDP treatment to modulate its distribution, potentially reducing adverse effects in sensitive organs.

In this study, we investigated the protective efficacy of two approaches: competition with OCT2-mediated CDDP uptake using some already approved drugs, and rapid regulation of OCT2 activity through known transporter regulation pathways. These in vitro evaluations were conducted to assess the potential of protective approaches in minimizing CDDP-induced cellular toxicity.

## 2. Results

### Interaction of Selected Drugs with OCT Transport

**Figure 1:**
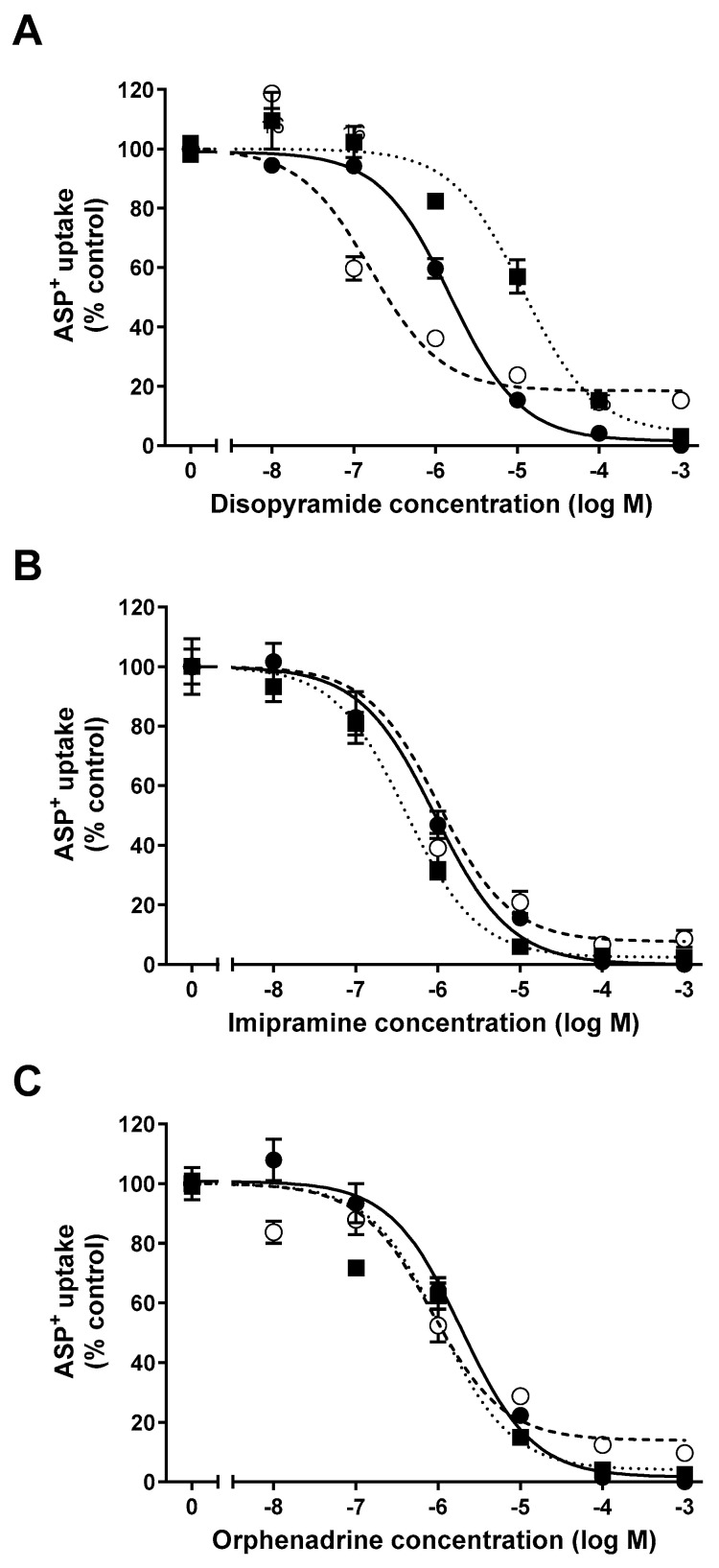
Effect of increasing concentrations of disopyramide (**panel A**), imipramine (**panel B**), or orphenadrine (**panel C**) on the uptake of 1 µM ASP^+^ in HEK293 stably expressing hOCT2 (closed circles, solid curve line), mOCT1 (open circles, dashed curve line), or mOCT2 (closed squares, dotted curve line). Results are expressed as mean ± SEM compared to what was measured in the absence of tested substance (=100%).

This study evaluated the interaction potential of the antiarrhythmic medication disopyramide, of the tricyclic antidepressant imipramine, and of the muscle relaxant orphenadrine with the transport of the fluorescent organic cation 4-(4-dimethylaminostyryl)-N-methylpyridinium (ASP^+^) in HEK293 cells stably expressing either the human OCT2 (hOCT2) or its murine ortholog and paralog OCT2 (mOCT2) and mOCT1. The mouse kidney is known to express both mOCT1 and mOCT2 in the basolateral membrane domain of proximal tubules [14]. The selection of disopyramide, imipramine, and orphenadrine for this study was based on their classification as organic cations, suggesting a potential interaction with OCTs [15,16]. Additionally, their existing approval and established safety profiles make them promising candidates for repurposing. Notably, disopyramide holds particular significance due to previous findings demonstrating its ability to alleviate CDDP nephrotoxicity in rats [17]. Conversely, imipramine presents an intriguing aspect, as it has been observed to inhibit the growth of neuroendocrine tumors [18]. This characteristic adds an interesting dimension to its potential therapeutic applications. As illustrated in Figure 1, it is evident that all the examined substances successfully inhibited the uptake of ASP^+^ in HEK cells stably expressing hOCT2, mOCT1, or mOCT2 in a concentration-dependent manner. Table 1 provides a summary of the IC_50_ values (the concentration of the substance that is required to inhibit the ASP^+^ uptake by 50%) measured in the interaction between disopyramide (Figure 1A), imipramine (Figure 1B), and orphenadrine (Figure 1C) with hOCT2, mOCT1, or mOCT2. These IC_50_ values are consistently within a similar concentration range. Nevertheless, noteworthy variations in the IC_50_ were observed specifically for the interaction of disopyramide with hOCT2, mOCT1, or mOCT2.

**Figure 2:**
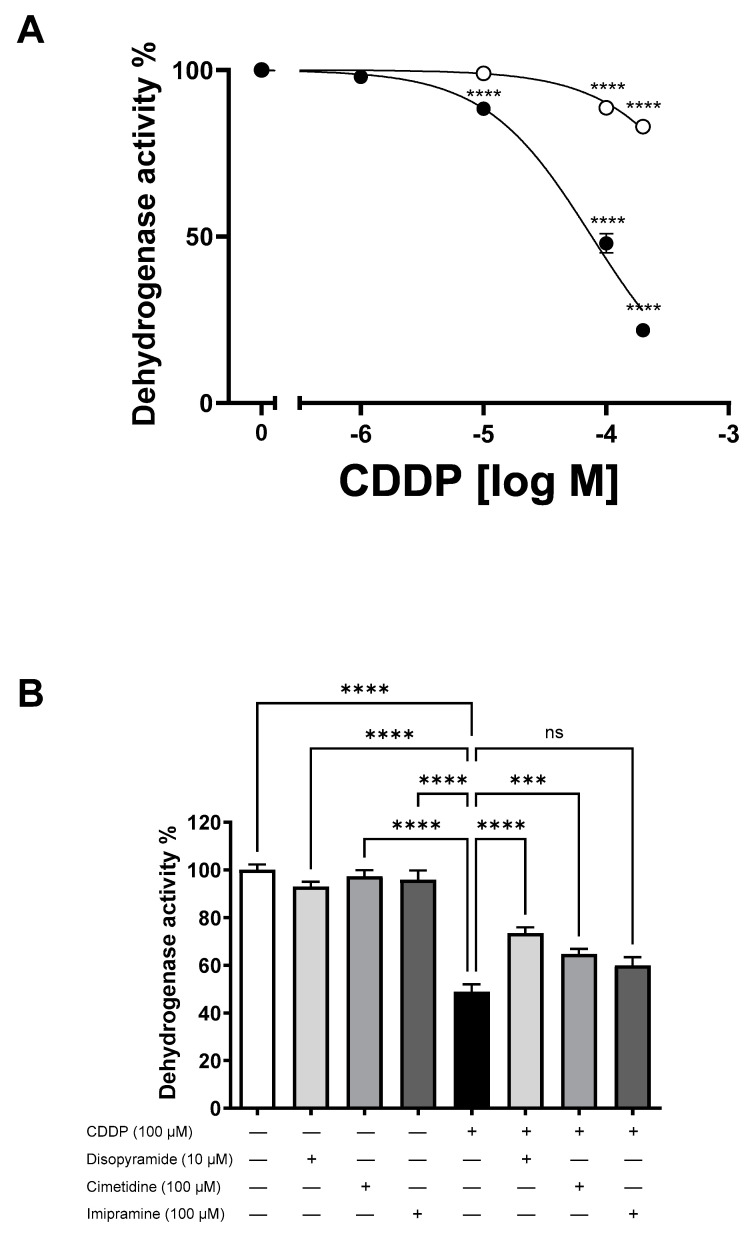
In (**Panel A**), the cell viability of both WT (open circles) and hOCT2-HEK293 (closed circles) cells is depicted in relation to increasing CDDP concentrations. This assessment was conducted by measuring dehydrogenase activity through an MTT assay. Cells were subjected to a 10 min incubation with a CDDP solution of the specified concentration. Subsequently, the incubation solution was removed, and the cells underwent a post-incubation period of 72 h in standard cell culture medium. The data, presented as mean ± SEM, are the result of 36 measurements across a minimum of three independent experiments. The asterisks (**** *p* < 0.0001) indicate a significant decrease in cell viability compared to control experiments without CDDP incubation, and for hOCT2-HEK293 cells, the asterisks indicate also a significant difference to experiments performed using the same CDDP concentration in WT-HEK293 cells, as determined by a 2-way ANOVA with Tukey’s multiple comparisons test. In (**Panel B**), the viability of HEK293 cells expressing hOCT2 is presented following a 10 min incubation with 100 µM CDDP alone or in combination with 10 µM disopyramide, 100 µM cimetidine, or 100 µM imipramine. This was followed by a post-incubation period of 72 h in normal CDDP-free cell culture medium. The data, represented as mean ± SEM, indicate a significant decrease in cell viability after treatment with 100 µM CDDP compared to control experiments, where cells were incubated for 10 min with cell culture medium without CDDP, with or without 10 µM disopyramide, 100 µM cimetidine, or 100 µM imipramine. Incubation with CDDP in conjunction with disopyramide or cimetidine significantly mitigated cellular CDDP toxicity (**** *p* < 0.0001 and *** *p* < 0.0003, ANOVA test, respectively). Co-incubation with 100 µM imipramine did not significantly decrease cellular CDDP toxicity (ns). The experiments involved a total of 5–12 replicates across a minimum of three independent experiments.

The comparative analysis of the cellular toxicity resulting from a 10 min incubation with escalating concentrations of CDDP (1–200 µM) in WT- and hOCT2-HEK293 cells was measured with an assay based on 3-(4,5-dimethylthiazol-2-yl)-2,5-diphenyltetrazolium bromide (MTT). This analysis revealed a significantly heightened sensitivity of hOCT2-HEK293 cells to CDDP treatment, which induced a significant cellular toxicity already at a 10 µM concentration, in contrast to what was observed in WT-HEK293 cells (Figure 2A). The toxicities measured at 100 and 200 µM CDDP were significantly higher in hOCT2- than in WT-HEK293 cells (Figure 2A). The calculated EC_50_ values (the EC_50_ value is the concentration that is required to obtain a 50% CDDP toxic effect) were 76 µM and 917 µM for hOCT2- and WT-HEK293 cells, respectively. Given the previously observed protective effect of disopyramide against CDDP-induced nephrotoxicity in rats [17], this study further investigated whether OCT2 might be a molecular mediator of this effect. To explore this, CDDP cytotoxicity was assessed in hOCT2-HEK293 cells with or without disopyramide using an MTT assay. In further experiments, protection by imipramine co-incubation was also tested. Disopyramide and imipramine concentrations of 10 and 100 µM, respectively, were chosen, as these concentrations nearly completely inhibited ASP^+^ uptake by hOCT2, as depicted in Figure 1A,B. In selected experiments, 100 µM cimetidine was employed as a protective substance, leveraging its demonstrated efficacy in reducing CDDP cellular toxicity mediated by CDDP transport via hOCT2 [8]. Exposure of hOCT2-HEK293 cells to a CDDP concentration of 100 µM for 10 min resulted in notable cellular toxicity, which was significantly mitigated through co-incubation with either 10 µM disopyramide or 100 µM cimetidine, but not with 100 µM imipramine, as illustrated in Figure 2B.

**Figure 3:**
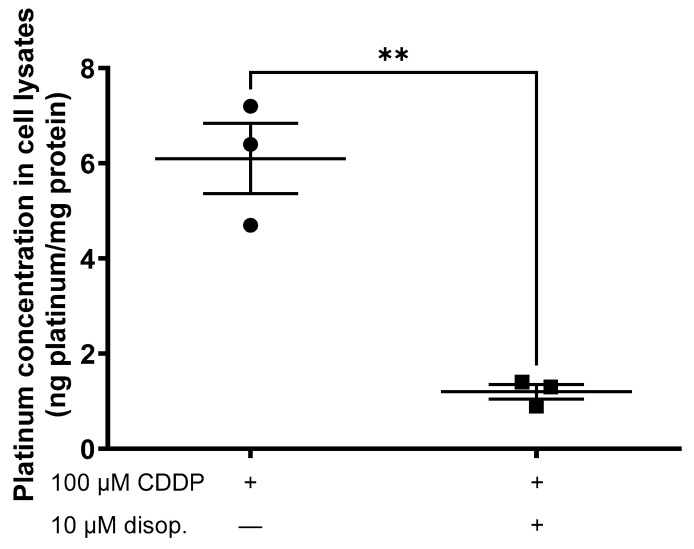
Inductively coupled plasma mass spectrometry (ICP-MS) analysis of platinum concentration in lysates from hOCT2-expressing HEK293 cells incubated for 10 min with 100 µM CDDP alone (closed circles) or together with 10 µM disopyramide (disop., closed squares). The results of the single experiments together with the mean± SEM are reported in the illustration. ** indicates a statistically significant difference (*p* = 0.003, unpaired *t*-test).

The assessment of the CDDP cellular accumulation in hOCT2-HEK293 cells following a 10 min incubation with 100 µM CDDP, either with or without 10 µM disopyramide, unequivocally demonstrated that the presence of dispopyramide caused a significant reduction in CDDP uptake (Figure 3).

To investigate the potential protection of polarized epithelial kidney cells against undesirable CDDP toxicity by regulation of hOCT2 activity, Madin–Darby Canine Kidney (MDCK) cells stably expressing hOCT2 (hOCT2-MDCK) were employed. The selection of MDCK cells was motivated by the intention to explore hOCT2 regulation in an environment closely resembling the physiological situation. In the renal cells of proximal tubules, characterized by distinct apical and basolateral membrane domains, hOCT2 is expressed in the basolateral membrane domain [19]. Following expression in MDCK cells, which are recognized for their morphological and functional polarization [20], hOCT2 exhibits a clear localization in the basolateral membrane domain [21]. This localization pattern in the basolateral membrane domain, as previously demonstrated, serves as a crucial factor in determining the nature of the hOCT2 regulation [21]. In this study, MDCK cells were cultured in the presence of an extracellular matrix to induce the formation of three-dimensional (3D) structures, known as cysts. Within these cysts, hOCT2 is unambiguously localized in the basolateral membrane domain, allowing for free access to experimental solutions [21].

**Figure 4:**
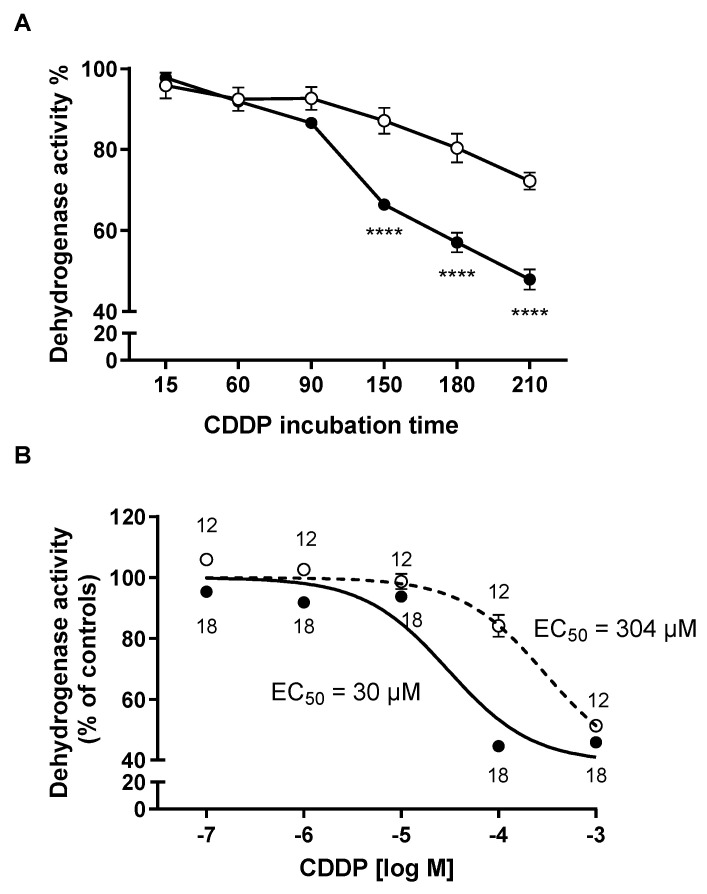
In this figure, the sensitivity of EV (open circles)- and hOCT2 (closed circles)-MDCK cysts to CDDP toxicity is depicted, measured as dehydrogenase activity through an MTT assay. (**Panel A**) presents the viability of EV (open circles)- and hOCT2 (closed circles)-MDCK cysts after incubation with 100 µM CDDP for the specified durations, followed by post-incubation periods of 24 h in normal CDDP-free cell culture medium. Under these conditions, starting from 150 min incubation, CDDP induced significantly stronger toxicity in MDCK cysts expressing hOCT2 than in EV-MDCK cysts (**** *p* < 0.0001, determined by a 2-way ANOVA with Tukey’s multiple comparisons test). The data are presented as mean ± SEM, and the experiments involved a total of 5 measurements in three independent experiments. In (**Panel B**), the sensitivity of EV (open circles)- and hOCT2 (closed circles)-MDCK cysts to toxicity induced by a 150 min incubation with increasing CDDP concentrations (0.1–1000 µM) is presented. This was followed by post-incubation periods of 24 h in normal CDDP-free cell culture medium, with dehydrogenase activity measured by an MTT assay. The impact of CDDP on cell viability was more pronounced in MDCK cysts expressing hOCT2 compared to EV-MDCK cysts, as indicated by the lower EC_50_ value. The EC_50_ values were 30 µM (logEC_50_ = −4.52 ± 0.07 with 88 degrees of freedom -DF) and 304 µM (logEC_50_ = −3.51 ± 0.12 with 58 DF) in hOCT2- and EV-MDCK cysts, respectively. These experiments were conducted with a total of 18 and 12 replicates in hOCT2- and EV-MDCK cysts, respectively, measured in at least three independent experiments. The data are presented as mean ± SEM.

In contrast to the expression of the empty vector (EV-MDCK cells), the expression of hOCT2 leads to a heightened susceptibility to CDDP toxicity. This susceptibility becomes significant after a 150 min incubation with 100 µM CDDP, followed by a 24 h incubation period in a CDDP-free medium (Figure 4A). Figure 4B shows a significantly higher EC_50_ for CDDP in EV-MDCK cells compared to hOCT2-MDCK cells.

**Figure 5:**
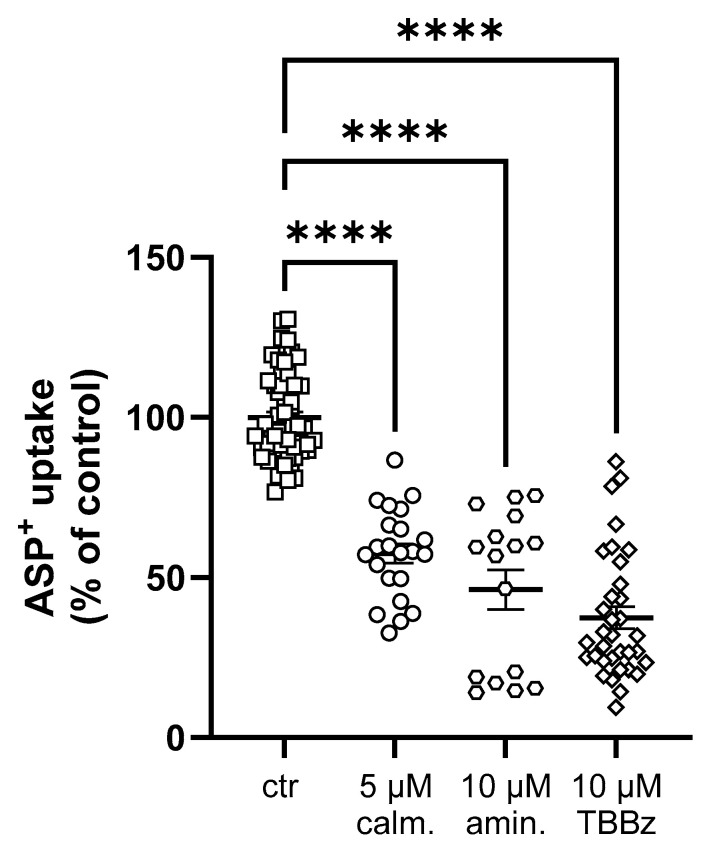
Rapid regulation of ASP^+^ uptake mediated by hOCT2 stably expressed in MDCK cysts (hOCT2- MDCK cysts). The regulation was investigated by inhibiting calmodulin with 5 µM calmidazolium (calm., open circles), p56*^lck^* tyrosine kinase with 10 µM aminogenistein (amin., open hexagons), and CKII with 10 µM TBBz (open diamonds). Initial uptake rates of ASP^+^ after 10 min incubation with these different inhibitors are presented as percentage of controls (ctr = 100%, open squares). Individual values with means ± SEM are displayed. A total of 16–55 replicates were measured in at least 3 independent experiments. The quadruple asterisks (****) indicate a statistically significant difference compared to control experiments (*p* < 0.0001, Brown–Forsythe and Welch ANOVA test).

In the 3D culture, hOCT2 activity has been demonstrated to be rapidly regulated by the p56*^lck^* tyrosine kinase, calmodulin, and casein kinase II (CKII) [21]. In this work, this regulation was confirmed, as shown in Figure 5, where the ASP^+^ uptake in hOCT2-MDCK cysts was measured after a 10 min incubation with 5 µM calmidazolium (calmodulin inhibitor), 10 µM aminogenistein (p56*^lck^* tyrosine kinase inhibitor), or 10 µM 4,5,6,7-tetrabromobenzimidazole (TBBz, CKII inhibitor).

**Figure 6:**
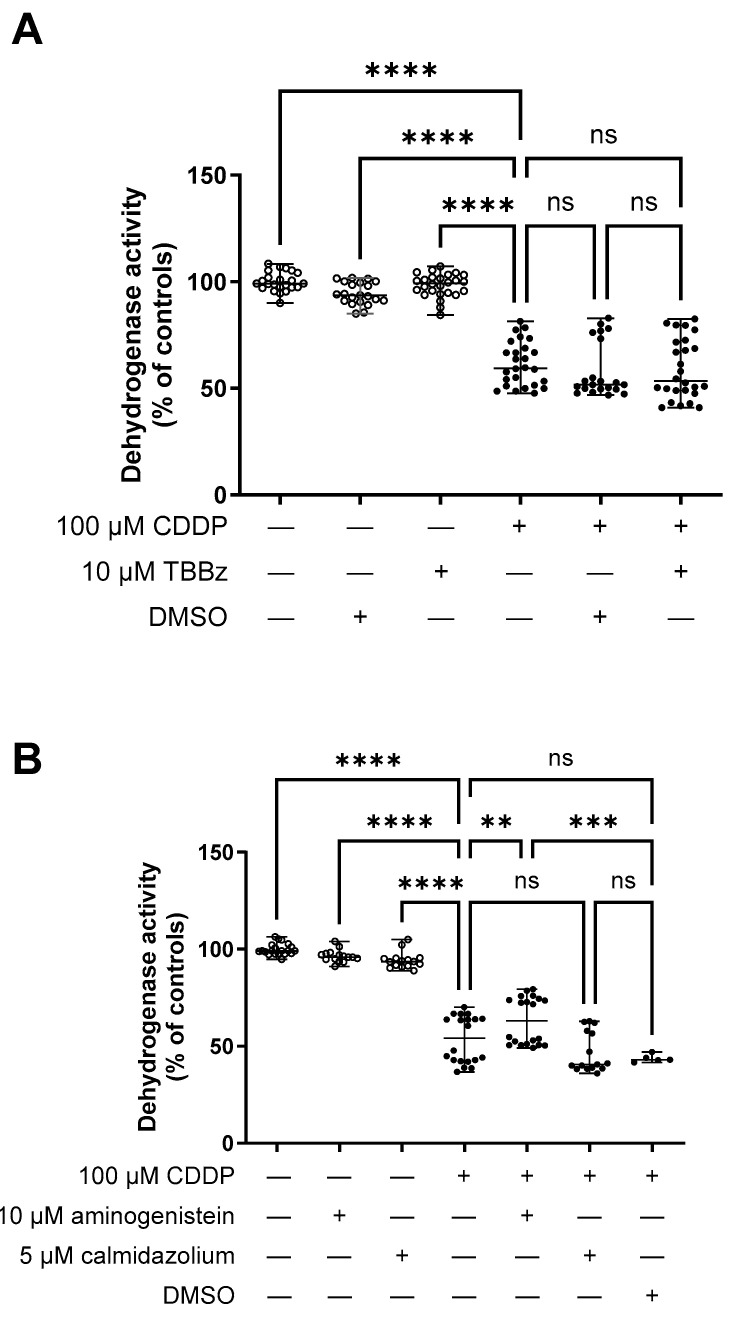
This figure shows the effect of calmodulin, p56*^lck^* tyrosine kinase, and CKII inhibition on CDDP toxicity in hOCT2-MDCK cysts measured as dehydrogenase activity by an MTT assay. Cells were treated with 100 µM CDDP in the presence or absence of a hOCT2 regulator for 150 min followed by 24 h post-incubation in normal CDDP-free cell culture medium (closed circles). The effects of the regulator alone and of the solvent (DMSO) on cell toxicity were also measured (open circles). (**Panel A**) shows the effect of TBBz addition (10 µM) to CDDP (100 µM) on cellular CDDP toxicity. Results are presented as median value with variation range. CDDP caused a significant cellular toxicity (**** *p* < 0.0001, determined by ANOVA test), which remained unchanged under additional incubation with TBBz. The presence of DMSO did not influence cell viability, nor CDDP toxicity. (**Panel B**) shows the effect of aminogenistein (10 µM) or calmidazolium (5 µM) addition to CDDP (100 µM) on cellular CDDP toxicity. Results are presented as median value with variation range. CDDP caused a significant cellular toxicity, which remained unchanged under additional incubation with calmidazolium but was significantly decreased under aminogenistein addition (**** *p* < 0.0001, *** *p* = 0.0003, ** *p* = 0.01, determined by ANOVA test, respectively). The presence of aminogensitein or calmidazolium alone did not change cell viability, and DMSO did not influence CDDP toxicity. Experiments were performed with a total of 5 replicates in 4–5 independent experiments.

Co-treatment with CDDP and TBBz (Figure 6A) or calmidazolium (Figure 6B) demonstrated no alteration in the CDDP cellular toxicity. Only the co-treatment with CDDP and aminogenistein exhibited a slight but significant amelioration of CDDP toxicity in the hOCT2-MDCK cells (Figure 6B).

## 3. Discussion

Owing to its demonstrated efficacy, a chemotherapy regime involving CDDP remains a primary choice for the treatment of newly diagnosed solid cancers [22]. This is notably evident in testicular cancer, where the combination of CDDP with surgery is regarded as a curative approach [23]. Nevertheless, the administration of CDDP is linked to the development of unwanted toxicities, including nephro- and ototoxicity. Despite the implementation of hydration protocols, CDDP-induced nephrotoxicity presents itself as acute or, in certain instances, chronic kidney injury, leading to life-threatening impairment of kidney function and a need for inclusion into surveillance programs. The prevalence of CDDP-induced nephrotoxicity ranges from 15 to 49%, depending on the study [24,25,26] and on the age of the patients [27,28]. Early indications of CDDP nephrotoxicity include a rapid reduction in the renal plasma flow and an elevation in urinary enzymes, which is characteristic of primary tubular toxicity. Such manifestations of renal injury are often concomitant with a Fanconi syndrome-like electrolyte disbalance such as hypomagnesemia, hypocalcemia, and hypokalemia, along with a decrease in the glomerular filtration rate (GFR) [29]. The severity of these effects is contingent on the administered doses of CDDP, with higher doses resulting in more pronounced manifestations. Patients treated with high doses of CDDP often exhibit proteinuria of glomerular and/or tubular origin, along with the described tubular dysfunction. Notably, these adverse effects can persist for up to 2 years after the termination of treatment [30]. CDDP chemotherapy is also associated with the development of ototoxicity. Ototoxicity is a clinical challenge, particularly in infants and younger children, where it poses a significant risk of delayed language development. This delay results from an impaired perception of higher-frequency consonant sounds due to damage of hair cells in the basal turn of the Corti organ, which is crucial for speech comprehension. Additionally, the spiral ganglion, spiral ligament, and cells of the stria vascularis undergo apoptosis as a result of CDDP exposure [31]. In challenging acoustic environments, such as those with background noise, speech comprehension is notably compromised. Consequently, school-age children may experience increased listening effort, concentration difficulties, and school challenges [32]. This ototoxicity is observed in over 50% of children who are treated with CDDP and can have devastating consequences for a young child’s social and educational development [33].

Due to the significant adverse effects associated with CDDP, alternative platinum derivatives, such as carboplatin and oxaliplatin, have been developed. Nonetheless, despite these alternatives, CDDP remains the preferred drug for the treatment of several solid malignancies due to its efficacy.

CDDP is excreted through the kidneys, where it interacts with the renal secretion pathway for organic cations [8,11,34], which in the basolateral membrane domain of renal proximal tubules is represented mainly by the organic cation transporters. In humans, this is primarily represented by hOCT2, while in mice, it involves mOCT1 and −2 [35,36]. OCTs are also present in other cells, such as those of the cochlea, and may contribute to CDDP ototoxicity [10]. CDDP uptake in cancer cells appears to be primarily facilitated by copper transporters [37,38]. However, it is worth noting that exceptions may exist, such as in colorectal cancer, where hOCT1 but not hOCT2 was detected [39]. Therefore, during CDDP treatment, strategies such as using competition of CDDP uptake mediated by OCTs with other organic cations or down-regulation of OCT activity could be valid approaches to reduce its toxicity. Given the well-established interaction of OCTs with various drugs that fall under the category of organic cations [40], repurposing such drugs could be a practicable option for achieving targeted organ protection against the undesired toxicity that is associated with CDDP. The C_max_ (the maximum serum concentrations) observed in patients for disopyramide, imipramine, and orphenadrine were reported to be 7.95, 0.71, and 0.31 µM, respectively [41]. Considering the IC_50_ for the inhibition of ASP^+^ uptake by disopyramide (see Table 1), it is evident that this value is well below the reported C_max_. This suggests that under therapeutic doses, disopyramide can attain concentrations in bodily fluids that are sufficient to effectively block hOCT2 activity, potentially safeguarding kidney and ear cells against CDDP toxicity. Conversely, IC_50_ for orphenadrine is above the reported C_max_, and for this, orphenadrine was no further investigated. Contrary to the observations made with 10 µM disopyramide, a concentration of 100 µM imipramine did not result in a significant rescue of cell viability under treatment with 100 µM CDDP (Figure 2B). For these reasons, our investigation specifically focused on the interaction of disopyramide with hOCT2, and the interactions of imipramine and orphenadrine were not further pursued in this study. In the toxicity experiments, a high concentration of CDDP (100 µM) was employed. This choice was based on the experimental system, where this concentration induces substantial toxicity in cells (HEK293 and MDCK cells) stably expressing hOCT2, while not or at a significantly lower level affecting the cells that are transfected with the empty vector (Figure 2A and Figure 4A). Moreover, in patients undergoing CDDP treatment, serum CDDP concentrations up to 25–40 µM can be detected [42,43].

This work demonstrates that disopyramide inhibits hOCT2 activity (Figure 1A), hOCT2-mediated CDDP toxicity (Figure 2B), and cellular accumulation (Figure 3). Therefore, these findings can explain the protection against CDDP nephrotoxicity by disopyramide co-treatment that have been observed in rats [17]. A notable difference in interaction potency was observed when examining the inhibition of the ASP^+^ uptake by disopyramide in HEK293 cells stably expressing mOCT1 or mOCT2 (Figure 1A). This finding suggests that in in vivo experiments involving mice, the concentration of disopyramide should be adjusted accordingly to specifically inhibit mOCT1 or mOCT2 activities, or both.

Regarding renal protection, it has been demonstrated that CDDP is secreted into the urine through the coordinated action of hOCT2 as an uptake transporter and Multidrug and Toxin Extrusion proteins (MATEs) 1 (MATE1) and 2k (MATE2k) as excretion transporters [44,45]. Importantly, disopyramide, imipramine, and orphenadrine exhibit much lower affinity for MATEs than for hOCT2 [41]. This observation suggests that the inhibition of the hOCT2-mediated CDDP uptake by these substances does not interfere with CDDP efflux into the urine by MATEs. However, a 100 µM concentration of imipramine significantly inhibits the ASP^+^ uptake mediated by hMATE1, while a 100 µM concentration of disopyramide has no significant effect. Since HEK293 endogenously expresses hMATE1, it can be supposed that inhibition of CDDP cellular efflux mediated by hMATE1 compromises the protective capacity of imipramine against CDDP toxicity in HEK293 cells.

The specific acute regulation of hOCT2 under CDDP treatment represents another potential option to decrease CDDP accumulation in kidneys and ears. However, this approach has not yet been investigated. In this study, the protection against CDDP cellular toxicity by hOCT2 regulation was examined in MDCK cells cultivated in an extracellular matrix. This cell system forms distinct apical and basolateral membrane domains [46], where hOCT2 is clearly localized in the basolateral membrane domains [21]. The sorting of transport proteins into the appropriate membrane domain is a tightly regulated process [47], potentially leading to different regulation pathways compared to non-polarized cell models [21]. In this work, acute regulation of hOCT2 by inhibiting CKII, p56*^lck^* tyrosine kinase, or calmodulin was investigated. These regulation pathways have previously been demonstrated to impact hOCT2 activity [21], and this influence was confirmed in the present work (Figure 5). It should be noted that the inhibition of CKII and of calmodulin but not of p56*^lck^* tyrosine kinase was shown to decrease cellular efflux of hMATE1 substrates [48]. Therefore, because of the endogenous expression of hMATE1 in HEK293 cells, the lack of protection by maneuvers on CKII and calmodulin may be caused by a reduction in hMATE1-mediated cellular CDDP detoxification. Specifically, only the inhibition of p56*^lck^* tyrosine kinase with aminogenistein, leading to a reduction in hOCT2 activity, was able to decrease the CDDP cellular toxicity (Figure 6B). The p56*^lck^* tyrosine kinase is a src-family tyrosine kinase, which is important for the function of T-cells [49] and which is known to phosphorylate a broad spectrum of downstream targets, thereby regulating diverse cellular functions, including cell movement, cell cycle progression, metabolism, cell-to-cell interactions, morphology, protein synthesis, and gene expression [50]. The multifaceted nature of p56*^lck^*’s phosphorylation activity underscores its pivotal role in coordinating various cellular processes. Interestingly, the activity of p56*^lck^* tyrosine kinase is also involved in the motility control of breast cancer cells [51] and can be a driver for metastatic processes in oral cancer [52]. Therefore, p56*^lck^* tyrosine kinase is a druggable kinase in cancer, and its inhibition may be a treatment option for some types of cancer and at the same time may reduce undesired effects of CDDP chemotherapy.

Therefore, besides “classical” competition approaches, the regulation approach could also help in decreasing CDDP cellular toxicity. A possible problem associated with this protection strategy is the specificity of regulation: probably, these regulatory pathways have important functions in the body. A possible targeting to the kidneys may be obtained by coupling regulators to some carrier, which then specifically delivers the regulators to the proximal tubule cells, as proposed in [53].

In conclusion, targeting hOCT2 as a CDDP transporter by competition or regulation approaches is at least in vitro a feasible treatment to diminish unwanted CDDP cellular toxicity.

## 4. Materials and Methods

### 4.1. Cell Lines

HEK293 cells stably expressing hOCT2, mOCT1, or mOCT2 were used in the experiments. The establishment of these cell lines has already been described in [54,55]. Cells were maintained in Dulbecco’s modified Eagle’s medium (DMEM; Biochrom, Berlin, Germany) supplemented with 3.7 g/L NaHCO_3_, 1.0 g/L d-glucose, 2.0 mM L-glutamine, 100 U/L penicillin, 100 mg/L streptomycin (Biochrom), 10% fetal bovine serum, and 0.8 mg/mL Geneticin (PAA Laboratories, Coelbe, Germany) at 37 °C in an 5% CO_2_ atmosphere. Cells within passages 15–80 were plated onto Nunclon 96 Flat bottom microtiter plates (Nunc, Wiesbaden, Germany) and allowed to reach confluence over a period of 2–3 days. The hOCT2 was expressed in MDCK II cells by viral transduction, as described in [21]. Generation of MDCK cells expressing hOCT2 (hOCT2-MDCK) or the empty vector alone (EV-MDCK) has already been described [21]. MDCK cells were cultured in Minimal Essential Medium Eagle (MEM, Sigma/Merck, Darmstadt, Germany) containing 10% FCS, 2 mM L-glutamine, and 1% penicillin/streptomycin. For obtaining a system where the basolateral plasma membrane domain was accessible to experimentation, 100 µL of an MDCK II cell suspension (150,000 cells/mL in MDCK II cell culture medium containing 2.5% Cultrex Basement Membrane Extract (BME2, Trevigen, Gaithersburg, MD, USA)) were seeded in a 96-well plate (Greiner, Frickenhausen, Germany), which was previously coated with 2 µL of ice-cooled BME2, and cultured at 37 °C with 5% CO_2_. On the third day of culture, the cell culture medium was refreshed. This method of cell culture within an extracellular matrix is commonly referred to as cyst-forming 3D culture [46], and for hOCT2-MDCK and EV-MDCK cells, this has already been described in detail in [21]. In the subsequent text, MDCK II cells expressing hOCT2-GFP or GFP alone and cultured in BME2 are denoted as hOCT2-MDCK cysts and empty vector (EV)-MDCK cysts, respectively.

Culture and functional analyses of these cells were approved by the state government Landesumweltamt Nordrhein-Westfalen, Essen, Germany (no. 521.-M-1.14/00).

### 4.2. Evaluation of OCT Function

The fluorescent organic cation ASP^+^ (Thermo Fisher Scientific, Waltham, MA, USA) served as the indicator of hOCT2 activity [55]. ASP^+^ is a well-established fluorescent substrate of OCT, as evidenced by numerous publications (see for example [21,56,57,58,59,60]). Fluorescence measurements were conducted using a microfluorescence plate reader (Infinite F200, Tecan Group Ltd., Crailsheim, Germany) with excitation at 450 nm and emission at 590 nm [56]. Transport measurements were carried out at a temperature of 37 °C. Prior to measurements, cell monolayers were rinsed with a Ringer-like solution composed of (in mM) NaCl 145, K_2_HPO_4_ 1.6, KH_2_PO_4_ 0.4, D-glucose 5, MgCl_2_ 1, and calcium gluconate 1.3, and with pH adjusted to 7.4 at 37 °C.

The apparent affinity (IC_50_) for inhibition of hOCT2-mediated ASP^+^ (1 µM) uptake by disopyramide, imipramine, and orphenadrine was determined through inhibition experiments in the presence of increasing competitor concentrations (10^−8^–10^−3^ M), as described in detail in [56]. In subsequent experiments, the regulation of hOCT2-mediated transport in MDCK cysts was assessed by co-incubating ASP^+^ with TBBz (10 µM), calmidazolium (5 µM), or aminogenistein (10 µM) as inhibitors of CKII, calmodulin, or p56*^lck^* tyrosine kinase, as described in detail in [21]. Solvent (DMSO) at the concentrations used in the regulation experiments did not alter ASP^+^ uptake. Experiments were conducted with cells from passages 20–45.

### 4.3. CDDP Toxicity Test

CDDP (Teva Pharm, Ulm, Germany) cytotoxicity was assessed using a modified MTT test [61]. First, confluent hOCT2- and EV-HEK293 cells were incubated for 10 min with different CDDP concentrations (1–200 µM), followed by a 72 h post-incubation time in CDDP-free cell culture medium. Following this incubation period, cells were treated with 10 µL of an MTT (Sigma/Merck) solution containing 5 mg/mL of the dye for three hours at 37 °C. After MTT removal, cells were lysed using a solution consisting of 10% (*w*/*v*) sodium dodecyl sulfate and 40% (*v*/*v*) dimethylformamide. Lysates were then transferred to a new microtiter plate, and after 90 min, absorbance was measured at 570 nm using a multiplate reader (Tecan Infinite M200, Tecan Group Ltd., Crailsheim, Germany). Since at a 100 µM CDDP concentration, a clear difference in cell viability between hOCT2- and EV-HEK293 cells was observed, further experiments were performed using this CDDP concentration. For protection experiments, CDDP incubation was performed in the presence or not of 10 µM disopyramide or 100 µM imipramine, and cell viability was measured after 72 h post-incubation in CDDP-free solution. When applying the MTT test in MDCK cysts, EV- and hOCT2-MDCK cysts were incubated with 100 µM CDDP for variable times (15–210 min), which were followed by 24 h post-incubation in CDDP-free cell culture medium and, finally, by an MTT test. Incubation times equal to or longer than 150 min resulted in a significant difference in cell viability between EV- and hOCT2-MDCK cysts. Therefore, protection experiments, where 10 µM TBBz, 5 µM calmidazolium, or 10 µM aminogenistein were added to CDDP, were performed using a CDDP incubation time of 150 min, followed by 24 h post-incubation in CDDP-free cell culture medium and, finally, by an MTT test.

### 4.4. Measurement of Intracellular CDDP Amount

The CCDP amount in hOCT2-HEK293 cells after 10 min incubation with 100 µM CDDP in the presence or not of 10 µM disopyramide was measured via inductively coupled plasma isotope dilution mass spectrometry (ICP-MS), as described in [62]. Briefly, after incubation with CDDP or CDDP with disopyramide, cells were washed 3 times with 1 mL ice-cold phosphate-buffered solution (PBS) before adding distilled water containing 4.2 ng/mL ^194^Pt as internal standard (Isoflex, San Francisco, CA, USA; certification number 78-02-194-4221) for hypoosmotic lysis. Lysates were placed in ultrasound bath on ice for 10 min and then centrifuged for 10 min at 13,000× *g*. The supernatant was used for platinum quantification and protein concentration measurement by a Bradford assay [63]. ICP-MS was conducted on a quadrupole mass analyzer system (iCAP Qc, Thermo Fisher Scientific, Bremen, Germany) equipped with a flatapole collision/reaction cell (QCell) coupled to a liquid chromatography (LC) device. The LC device comprised an AccelaPump (model 1000 or 1250) for delivering the carrier solution and an AccelaAutosampler, both from Thermo Fisher Scientific. No column was mounted on the LC device, which served as the injection device for the flow injection analysis. The sample introduction system of the quadrupole instrument included a Peltier-cooled cyclonic quartz glass spray chamber operated at 2.7 °C, a μFlow ST nebulizer, a quartz injector pipe (ID = 1.8 mm), and Ni sampler and skimmer cones, the latter with a 2.8 mm insert. The collision/reaction gas within the flatapole consisted of a mixture of 8% (*v*/*v*) H_2_ in He (purity 99.999%). The instrument was operated using Qtegra 1.5 (Thermo Fisher Scientific) and tuned daily according to the manufacturer’s recommendations. This software was also employed for peak integration using the interactive chemical information system (ICIS) peak identification and integration algorithm.

### 4.5. Chemicals

All chemicals used in the study were of the highest purity and sourced from Sigma/Merck, unless specified otherwise.

### 4.6. Statistical Analysis

When not otherwise specified, data in this study are presented as mean values ± SEM. Statistical analyses, including unpaired t-tests and ANOVA tests, were performed using GraphPad Prism, Version 10.0 (GraphPad Software, Inc., San Diego, CA, USA), with a significance level set at *p* < 0.05. (*n*) denotes the number of replicates, and *N* denotes the number of independent experiments. IC_50_ and EC_50_ values were derived through non-linear sigmoidal concentration–response curve fitting and compared using GraphPad Prism, Version 10.0.

## Figures and Tables

**Table 1:** IC_50_ values for inhibition of ASP^+^ uptake by disopyramide, imipramine, and orphenadrine (all values given as µM), determined using hOCT2-, mOCT1, or mOCT2-HEK cells. The logIC_50_ values ± SEM and the number of replicates (*n*) measured in at least 3 independent experiments are also indicated.

	IC_50_ (logIC_50_ ± SEM) (µM)		
Cell Line/Substance	Disopyramide	Imipramine	Orphenadrine
hOCT2-HEK	1.5 * (−5.82 ± 0.06) *n* = 15–44	0.9 (−6.05 ± 0.09) *n* = 8–38	2.0 (−5.72 ± 0.08) *n* = 16–70
mOCT1-HEK	0.2 * (−6.79 ± 0.12) *n* = 8–31	1.0 (−5.99 ± 0.30) *n* = 8–48	0.8 (−6.08 ± 0.13) *n* = 8–47
mOCT2-HEK	11.8 * (−4.93 ± 0.09) *n* = 8–32	0.4 (−6.39 ± 0.06) *n* = 8–28	1.0 (−6.00 ± 0.07) *n* = 16–56

* indicates a statistically significant difference from what was calculated in the other cell lines for disopyramide (* *p* < 0.0001, ANOVA test).

## Data Availability

Data is contained within the article.

## References

[1] Bai Y., Aodeng G., Ga L., Hai W., Ai J. (2023). Research Progress of Metal Anticancer Drugs. Pharmaceutics.

[2] Ghosh S. (2019). Cisplatin: The First Metal Based Anticancer Drug. Bioorg. Chem..

[3] Ahmad S. (2017). Kinetic Aspects of Platinum Anticancer Agents. Polyhedron.

[4] Ratanaphan A., Kruman I. (2011). A DNA Repair Protein BRCA1 as a Potentially Molecular Target for the Anticancer Platinum Drug Cisplatin. DNA Repair.

[5] Minerva, Bhat A., Verma S., Chander G., Jamwal R.S., Sharma B., Bhat A., Katyal T., Kumar R., Shah R. (2023). Cisplatin-Based Combination Therapy for Cancer. J. Cancer Res. Ther..

[6] Ruano L., Cárdenas G., Nogueira J.J. (2021). The Permeation Mechanism of Cisplatin Through a Dioleoylphosphocholine Bilayer. Chem. Phys. Chem..

[7] Ishida S., Lee J., Thiele D.J., Herskowitz I. (2002). Uptake of the Anticancer Drug Cisplatin Mediated by the Copper Transporter Ctr1 in Yeast and Mammals. Proc. Natl. Acad. Sci. USA.

[8] Ciarimboli G., Ludwig T., Lang D., Pavenstädt H., Koepsell H., Piechota H.-J., Haier J., Jaehde U., Zisowsky J., Schlatter E. (2005). Cisplatin Nephrotoxicity Is Critically Mediated via the Human Organic Cation Transporter 2. Am. J. Pathol..

[9] Kim E.S., Tang X., Peterson D.R., Kilari D., Chow C.-W., Fujimoto J., Kalhor N., Swisher S.G., Stewart D.J., Wistuba I.I. (2014). Copper Transporter CTR1 Expression and Tissue Platinum Concentration in Non-Small Cell Lung Cancer. Lung Cancer.

[10] Ciarimboli G., Deuster D., Knief A., Sperling M., Holtkamp M., Edemir B., Pavenstädt H., Lanvers-Kaminsky C., am Zehnhoff-Dinnesen A., Schinkel A.H. (2010). Organic Cation Transporter 2 Mediates Cisplatin-Induced Oto- and Nephrotoxicity and Is a Target for Protective Interventions. Am. J. Pathol..

[11] Yonezawa A., Masuda S., Nishihara K., Yano I., Katsura T., Inui K. (2005). Association between Tubular Toxicity of Cisplatin and Expression of Organic Cation Transporter ROCT2 (Slc22a2) in the Rat. Biochem. Pharmacol..

[12] Sprowl J.A., Lancaster C.S., Pabla N., Hermann E., Kosloske A.M., Gibson A.A., Li L., Zeeh D., Schlatter E., Janke L.J. (2014). Cisplatin-Induced Renal Injury Is Independently Mediated by OCT2 and P53. Clin. Cancer Res..

[13] Sprowl J.A., van Doorn L., Hu S., van Gerven L., de Bruijn P., Li L., Gibson A.A., Mathijssen R.H., Sparreboom A. (2013). Conjunctive Therapy of Cisplatin with the OCT2 Inhibitor Cimetidine: Influence on Antitumor Efficacy and Systemic Clearance. Clin. Pharmacol. Ther..

[14] Basit A., Radi Z., Vaidya V.S., Karasu M., Prasad B. (2019). Kidney Cortical Transporter Expression across Species Using Quantitative Proteomics. Drug Metab. Dispos..

[15] Zhou S., Zeng S., Shu Y. (2021). Drug-Drug Interactions at Organic Cation Transporter 1. Front. Pharmacol..

[16] Bönisch H. (2021). Substrates and Inhibitors of Organic Cation Transporters (OCTs) and Plasma Membrane Monoamine Transporter (PMAT) and Therapeutic Implications. Handb. Exp. Pharmacol..

[17] Hanada K., Odaka K., Kudo A., Ogata H. (1999). Effects of Disopyramide and Verapamil on Renal Disposition and Nephrotoxicity of Cisplatin in Rats. Pharm. Res..

[18] Jahchan N.S., Dudley J.T., Mazur P.K., Flores N., Yang D., Palmerton A., Zmoos A.-F., Vaka D., Tran K.Q.T., Zhou M. (2013). A Drug Repositioning Approach Identifies Tricyclic Antidepressants as Inhibitors of Small Cell Lung Cancer and Other Neuroendocrine Tumors. Cancer Discov..

[19] Motohashi H., Nakao Y., Masuda S., Katsura T., Kamba T., Ogawa O., Inui K.-I. (2013). Precise Comparison of Protein Localization among OCT, OAT, and MATE in Human Kidney. J. Pharm. Sci..

[20] Caceres P.S., Gravotta D., Zager P.J., Dephoure N., Rodriguez-Boulan E. (2019). Quantitative Proteomics of MDCK Cells Identify Unrecognized Roles of Clathrin Adaptor AP-1 in Polarized Distribution of Surface Proteins. Proc. Natl. Acad. Sci. USA.

[21] Koepp T.N., Tokaj A., Nedvetsky P.I., Conchon Costa A.C., Snieder B., Schröter R., Ciarimboli G. (2021). Properties of Transport Mediated by the Human Organic Cation Transporter 2 Studied in a Polarized Three-Dimensional Epithelial Cell Culture Model. Int. J. Mol. Sci..

[22] Brown A., Kumar S., Tchounwou P.B. (2019). Cisplatin-Based Chemotherapy of Human Cancers. J. Cancer Sci. Ther..

[23] Einhorn L.H. (2002). Curing Metastatic Testicular Cancer. Proc. Natl. Acad. Sci. USA.

[24] Mwenda V., Githuku J., Gathecha G., Wambugu B.M., Roka Z.G., Ong’or W.O. (2019). Prevalence and Factors Associated with Chronic Kidney Disease among Medical Inpatients at the Kenyatta National Hospital, Kenya, 2018: A Cross-Sectional Study. Pan. Afr. Med. J..

[25] Isiiko J., Atwiine B., Oloro J. (2021). Prevalence and Risk Factors of Nephrotoxicity Among Adult Cancer Patients at Mbarara Regional Referral Hospital. Cancer Manag. Res..

[26] Mohri J., Katada C., Ueda M., Sugawara M., Yamashita K., Moriya H., Komori S., Hayakawa K., Koizumi W., Atsuda K. (2018). Predisposing Factors for Chemotherapy-Induced Nephrotoxicity in Patients with Advanced Esophageal Cancer Who Received Combination Chemotherapy with Docetaxel, Cisplatin, and 5-Fluorouracil. J. Transl. Int. Med..

[27] Duan Z., Cai G., Li J., Chen X. (2020). Cisplatin-Induced Renal Toxicity in Elderly People. Ther. Adv. Med. Oncol..

[28] Schofield J., Harcus M., Pizer B., Jorgensen A., McWilliam S. (2023). Long-Term Cisplatin Nephrotoxicity after Childhood Cancer: A Systematic Review and Meta-Analysis. Pediatr. Nephrol..

[29] Pabla N., Dong Z. (2008). Cisplatin Nephrotoxicity: Mechanisms and Renoprotective Strategies. Kidney Int..

[30] Daugaard G. (1990). Cisplatin Nephrotoxicity: Experimental and Clinical Studies. Dan. Med. Bull..

[31] Rybak L.P., Ramkumar V. (2007). Ototoxicity. Kidney Int..

[32] Rybak L., Mukherjea D., Ramkumar V. (2019). Mechanisms of Cisplatin-Induced Ototoxicity and Prevention. Semin. Hear..

[33] Skinner R. (2004). Best Practice in Assessing Ototoxicity in Children with Cancer. Eur. J. Cancer.

[34] Filipski K.K., Loos W.J., Verweij J., Sparreboom A. (2008). Interaction of Cisplatin with the Human Organic Cation Transporter 2. Clin. Cancer Res..

[35] Wright S.H., Dantzler W.H. (2004). Molecular and Cellular Physiology of Renal Organic Cation and Anion Transport. Physiol. Rev..

[36] Alnouti Y., Petrick J.S., Klaassen C.D. (2006). Tissue Distribution and Ontogeny of Organic Cation Transporters in Mice. Drug Metab. Dispos..

[37] Safaei R., Howell S.B. (2005). Copper Transporters Regulate the Cellular Pharmacology and Sensitivity to Pt Drugs. Crit. Rev. Oncol. Hematol..

[38] Safaei R. (2006). Role of Copper Transporters in the Uptake and Efflux of Platinum Containing Drugs. Cancer Lett..

[39] Zhang S., Lovejoy K.S., Shima J.E., Lagpacan L.L., Shu Y., Lapuk A., Chen Y., Komori T., Gray J.W., Chen X. (2006). Organic Cation Transporters Are Determinants of Oxaliplatin Cytotoxicity. Cancer Res..

[40] Koepsell H., Lips K., Volk C. (2007). Polyspecific Organic Cation Transporters: Structure, Function, Physiological Roles, and Biopharmaceutical Implications. Pharm. Res..

[41] Kido Y., Matsson P., Giacomini K.M. (2011). Profiling of a Prescription Drug Library for Potential Renal Drug-Drug Interactions Mediated by the Organic Cation Transporter 2. J. Med. Chem..

[42] Jehn C.F., Boulikas T., Kourvetaris A., Possinger K., Lüftner D. (2007). Pharmacokinetics of Liposomal Cisplatin (Lipoplatin) in Combination with 5-FU in Patients with Advanced Head and Neck Cancer: First Results of a Phase III Study. Anticancer. Res..

[43] Rajkumar P. (2016). Cisplatin Concentrations in Long and Short Duration Infusion: Implications for the Optimal Time of Radiation Delivery. J. Clin. Diagn. Res..

[44] Yonezawa A., Inui K.-I. (2011). Organic Cation Transporter OCT/SLC22A and H(+)/Organic Cation Antiporter MATE/SLC47A Are Key Molecules for Nephrotoxicity of Platinum Agents. Biochem. Pharmacol..

[45] Yonezawa A., Masuda S., Yokoo S., Katsura T., Inui K.-I. (2006). Cisplatin and Oxaliplatin, but Not Carboplatin and Nedaplatin, Are Substrates for Human Organic Cation Transporters (SLC22A1-3 and Multidrug and Toxin Extrusion Family). J. Pharmacol. Exp. Ther..

[46] Elia N., Lippincott-Schwartz J. (2009). Culturing MDCK Cells in Three Dimensions for Analyzing Intracellular Dynamics. Curr. Protoc. Cell Biol..

[47] Muth T.R., Caplan M.J. (2003). Transport Protein Trafficking in Polarized Cells. Annu. Rev. Cell Dev. Biol..

[48] Kantauskaitė M., Hucke A., Reike M., Ahmed Eltayeb S., Xiao C., Barz V., Ciarimboli G. (2020). Rapid Regulation of Human Multidrug and Extrusion Transporters HMATE1 and HMATE2K. Int. J. Mol. Sci..

[49] Harashima N., Tanaka K., Sasatomi T., Shimizu K., Miyagi Y., Yamada A., Tamura M., Yamana H., Itoh K., Shichijo S. (2001). Recognition of the Lck Tyrosine Kinase as a Tumor Antigen by Cytotoxic T Lymphocytes of Cancer Patients with Distant Metastases. Eur. J. Immunol..

[50] Rudd C.E. (2021). How the Discovery of the CD4/CD8-P56lck Complexes Changed Immunology and Immunotherapy. Front. Cell Dev. Biol..

[51] Mahabeleshwar G.H., Das R., Kundu G.C. (2004). Tyrosine Kinase, P56 -Induced Cell Motility, and Urokinase-Type Plasminogen Activator Secretion Involve Activation of Epidermal Growth Factor Receptor/Extracellular Signal Regulated Kinase Pathways. J. Biol. Chem..

[52] Weiße J., Rosemann J., Müller L., Kappler M., Eckert A.W., Glaß M., Misiak D., Hüttelmaier S., Ballhausen W.G., Hatzfeld M. (2021). Identification of Lymphocyte Cell-Specific Protein-Tyrosine Kinase (LCK) as a Driver for Invasion and Migration of Oral Cancer by Tumor Heterogeneity Exploitation. Mol. Cancer.

[53] Wischnjow A., Sarko D., Janzer M., Kaufman C., Beijer B., Brings S., Haberkorn U., Larbig G., Kübelbeck A., Mier W. (2016). Renal Targeting: Peptide-Based Drug Delivery to Proximal Tubule Cells. Bioconjug. Chem..

[54] Lee W.-K., Reichold M., Edemir B., Ciarimboli G., Warth R., Koepsell H., Thévenod F. (2009). Organic Cation Transporters OCT1, 2, and 3 Mediate High-Affinity Transport of the Mutagenic Vital Dye Ethidium in the Kidney Proximal Tubule. Am. J. Physiol. Renal Physiol..

[55] Schlatter E., Klassen P., Massmann V., Holle S.K., Guckel D., Edemir B., Pavenstädt H., Ciarimboli G. (2014). Mouse Organic Cation Transporter 1 Determines Properties and Regulation of Basolateral Organic Cation Transport in Renal Proximal Tubules. Pflugers Arch..

[56] Wilde S., Schlatter E., Koepsell H., Edemir B., Reuter S., Pavenstädt H., Neugebauer U., Schröter R., Brast S., Ciarimboli G. (2009). Calmodulin-Associated Post-Translational Regulation of Rat Organic Cation Transporter 2 in the Kidney Is Gender Dependent. Cell Mol. Life Sci..

[57] Salomon J.J., Endter S., Tachon G., Falson F., Buckley S.T., Ehrhardt C. (2012). Transport of the Fluorescent Organic Cation 4-(4-(Dimethylamino)Styryl)-N-Methylpyridinium Iodide (ASP+) in Human Respiratory Epithelial Cells. Eur. J. Pharm. Biopharm..

[58] Wittwer M.B., Zur A.A., Khuri N., Kido Y., Kosaka A., Zhang X., Morrissey K.M., Sali A., Huang Y., Giacomini K.M. (2013). Discovery of Potent, Selective Multidrug and Toxin Extrusion Transporter 1 (MATE1, SLC47A1) Inhibitors Through Prescription Drug Profiling and Computational Modeling. J. Med. Chem..

[59] Tzvetkov M.V., Saadatmand A.R., Bokelmann K., Meineke I., Kaiser R., Brockmöller J. (2012). Effects of OCT1 Polymorphisms on the Cellular Uptake, Plasma Concentrations and Efficacy of the 5-HT(3) Antagonists Tropisetron and Ondansetron. Pharmacogenomics J..

[60] Mayer F.P., Schmid D., Holy M., Yang J.-W., Salzer I., Boehm S., Chiba P., Sitte H.H. (2012). Real-Time Uptake of Fluorescent ASP+ via the Organic Cation Transporter 3. BMC Pharmacol. Toxicol..

[61] Mosmann T. (1983). Rapid Colorimetric Assay for Cellular Growth and Survival: Application to Proliferation and Cytotoxicity Assays. J. Immunol. Methods.

[62] Wehe C.A., Beyer G., Sperling M., Ciarimboli G., Karst U. (2014). Assessing the Intracellular Concentration of Platinum in Medulloblastoma Cell Lines after Cisplatin Incubation. J. Trace Elem. Med. Biol..

[63] Bradford M.M. (1976). A Rapid and Sensitive Method for the Quantitation of Microgram Quantities of Protein Utilizing the Principle of Protein-Dye Binding. Anal. Biochem..

